# Targeting methanotrophs and isolation of a novel psychrophilic* Methylobacter* species from a terrestrial Arctic alkaline methane seep in Lagoon Pingo, Central Spitsbergen (78° N)

**DOI:** 10.1007/s10482-024-01953-1

**Published:** 2024-03-22

**Authors:** Shalaka K. Patil, Tajul Islam, Alexander Tveit, Andrew Hodson, Lise Øvreås

**Affiliations:** 1 Department of Biological Sciences, University of Bergen, Postboks 7803, 5020 Bergen, Norway; 2 Department of Arctic and Marine Biology, The Arctic University of Tromsø, 9037 Tromsø, Norway; 3 University Centre in Svalbard, 9171 Longyearbyen, Norway; 4 Bjerknes Centre for Climate Research, Jahnebakken 5, 5007 Bergen, Norway

**Keywords:** Cold methane seeps, Methanotrophs, *Methylobacter*, Pingos, pMMO, Svalbard

## Abstract

The microbial diversity associated with terrestrial groundwater seepage through permafrost soils is tightly coupled to the geochemistry of these fluids. Terrestrial alkaline methane seeps from Lagoon Pingo, Central Spitsbergen (78°N) in Norway, with methane-saturated and oxygen-limited groundwater discharge providing a potential habitat for methanotrophy. Here, we report on the microbial community’s comparative analyses and distribution patterns at two sites close to Lagoon Pingo’s methane emission source. To target methane-oxidizing bacteria from this system, we analysed the microbial community pattern of replicate samples from two sections near the main methane seepage source. DNA extraction, metabarcoding and subsequent sequencing of 16S rRNA genes revealed microbial communities where the major prokaryotic phyla were *Pseudomonadota* (42–47%), *Gemmatimonadota* (4–14%) and *Actinobacteriota* (7–11%). Among the *Pseudomonadota*, members of the genus *Methylobacter* were present at relative abundances between 1.6 and 4.7%. Enrichment targeting the methane oxidising bacteria was set up using methane seep sediments as inoculum and methane as the sole carbon and energy source, and this resulted in the isolation of a novel psychrophilic methane oxidizer, LS7-T4A^T^. The optimum growth temperature for the isolate was 13 °C and the pH optimum was 8.0. The morphology of cells was short rods, and TEM analysis revealed intracytoplasmic membranes arranged in stacks, a distinctive feature for Type I methanotrophs in the family *Methylomonadaceae* of the class *Gammaproteobacteria*. The strain belongs to the genus *Methylobacter* based on high 16S rRNA gene similarity to the psychrophilic species of *Methylobacter psychrophilus* Z-0021^T^ (98.95%), the psychrophilic strain *Methylobacter* sp. strain S3L5C (99.00%), and the Arctic mesophilic species of *Methylobacter tundripaludum* SV96^T^ (99.06%). The genome size of LS7-T4A^T^ was 4,338,157 bp with a G + C content of 47.93%. The average nucleotide identities (ANIb) of strain LS7-T4A^T^ to 10 isolated strains of genus *Methylobacter* were between 75.54 and 85.51%, lower than the species threshold of 95%. The strain LS7-T4A^T^ represents a novel Arctic species, distinct from other members of the genus *Methylobacter*, for which the name *Methylobacter svalbardensis* sp. nov. is proposed. The type of strain is LS7-T4A^T^ (DSMZ:114308, JCM:39463).

**Supplementary Information:**

The online version contains supplementary material available at 10.1007/s10482-024-01953-1.

## Introduction

Arctic permafrost is considered critically climate sensitive because temperature increases lead to thaw and higher microbial activity, organic carbon degradation, and increased emissions of the greenhouse gases methane and carbon dioxide (Schuur et al. 2013). In the Arctic, much methane is released into the atmosphere through ice-cored permafrost hills (Hodson et al.^2019^). These dome-shaped landforms forming on permafrost due to artesian pressure are called open systems pingos (Liestøl, 1977; Gurney 1998; Grosse and Jones 2010). These landforms occur in the lowlands of mountainous cold regions from pressurized water emerging from deep underground (Hammock, et al. 2022). The pingos are ice-cored from the partial or complete freezing of upwelling groundwaters near the land surface (Demidov et al. 2022). When freezing is incomplete, pingos develop springs that discharge biogenic or thermogenic methane (Hodson et al. 2020). Such open system pingos are common in central Svalbard and highly dynamic (Hjelle 1993). They are often found in clusters and are significant sources of groundwater discharge (Gurney 1998). These ubiquitous permafrost-diagnostic landforms’ formations and their internal structure still remain unclear (Hammock et al. 2022). Still, little is known about the origin of the subsurface groundwater and the distribution of methane; however, carbon isotope composition indicates that thermogenic methane mixes with microbial biogenic methane below the permafrost (Hodson et al. 2020). The methane enters the atmosphere by degassing spring water through diffusion and ebullition or by venting directly.

In Adventdalen, four out of six pingo structures described are characterized by uninterrupted annual groundwater discharge, releasing approximately 1040 kg of CH_4_ into the atmosphere annually (Hodson et al. 2020). Lagoon pingo is the most studied open-pingo system in Adventdalen, making it a model site for understanding such dynamic systems (Orvin 1944; Svensson 1970; Liestol 1976; Yoshikawa 1993; Yoshikawa and Harada 1995; Yoshikawa and Nakamura 1996). Lagoon pingo is the youngest pingo system in Adventdalen, estimated to be about 160 ± 20 years old (Yoshikawa and Nakamura 1996), and is still active. It is situated close to Adventfjorden yet protected from the tides by Moskuslagunen and is composed by three crater ponds discharging groundwater enriched with methane (Hodson et al.^2019^). In methane-rich environments, two different types of biological methane oxidation occur depending on oxygen availability. Under anaerobic conditions, consortia of anaerobic methane-oxidizing archaea (ANME) and sulfate-reducing bacteria oxidize methane using sulfate as an electron acceptor. In contrast, under aerobic conditions, methane-oxidizing bacteria (MOB) or methanotrophs can utilize methane, either mixotrophically or as their sole source of carbon and energy, with oxygen as an electron acceptor (Knief 2015).

MOB constitute a ubiquitous group of bacteria that share the distinctive ability to metabolize methane (Hanson and Hanson 1996). So far, these bacteria are shown to be phylogenetically affiliated with the phyla of *Pseudomonadota* (Houghton et al. 2019), *Verrucomicrobia* (Camp et al. 2009; Islam et al. 2008; Dunfield et al. 2007), and *Actinobacteriota* (van Spanning et al. 2022). Most methanotrophic bacteria are mesophilic and neutrophilic organisms isolated from diverse extreme environments (Trotsenko and Khmelenina 2002). Psychrophilic, mesophilic and thermophilic methanotrophic bacteria are known to be found in distinct genera of the *Gammaproteobacteria* class (Knief 2015, 2019). MOBs are critical representatives in the CH_4_ cycle with a massive influence on CH_4_ fluxes. They are often found living at the oxic-anoxic interfaces in CH_4_-emitting ecosystems such as wetlands (Chowdhury and Dick 2013; Danilova et al. 2013; Danilova and Dedysh 2014), lakes (Bowman et al. 1997; Costello and Lindstrom (1999), marine sediments (Deutzmann et al. 2011; Dumont et al. 2011), landfills (Chen et al. 2007), bogs (Dedysh 2002; Dedysh et al. 2000; Belova et al. 2011, 2013) and rice fields (Knief 2015). Many MOBs, especially within *Gammaproteobacteria*, are obligate methanotrophs oxidizing CH_4_ for biomass formation and CO_2_ generation (Knief 2015), while others are facultative methylotrophs, capable of using other carbon and energy sources such as acetate, methanol, or ethanol (i.e. *Methylocystis* and *Methylocella*, (Dedysh et al. 2004; Vorobev et al. 2011; Im et al. 2011) and even gases such as H_2_ and CO_2_ (Tveit et al. 2019).

Two psychrophilic methanotrophic species, *Methylosphaera hansonii* (Bowman et al. 1997) and *Methylobacter psychrophilus* Z-0021^ T^ (Omelchenko et al. 1996), from the family *Methylomonadaceae* have been characterized and isolated from surface sediments of an Antarctic meromictic lake and from Russian Arctic tundra soil, respectively. Only very few genome sequences of psychrophilic methane oxidizers have been reported. Recently, the genome of the psychrophilic tundra soil strain *M. psychrophilus* Z-0021^T^ (DSM 9914) was sequenced (Rissanen et al. 2022). An obligate psychrophilic methanotroph in the genus *Methylobacter,* retrieved from a boreal lake in Finland, has also been characterized by full genome sequencing (Khanongnuch et al. 2022, 2023). Other psychrophilic or psychrotolerant isolates have been reported from tundra soil, permafrost environments, arctic wetlands, saline meromictic lakes, polar lakes, wet plant material, Arctic thermal spring water and sediments in maritime Antarctica (Trotsenko et al. 2005; Bowman et al. 1997; Wartiainen et al. 2006; Oshkin et al. 2016; Mateos-Rivera et al. 2018; Islam et al. 2020; Roldán and Menes 2023).

For the detection and diversity analysis of C_1_-utilizing bacteria, several functional marker genes are commonly used, such as *pmoA* (encoding a subunit of the particulate methane monooxygenase, pMMO: a copper-dependent enzyme), *mmoX* (encoding a subunit of the soluble methane monooxygenase, sMMO: an iron-dependent enzyme), *mxaF* (encoding the large subunit of PQQ-dependent methanol dehydrogenase, MDH: a calcium-containing enzyme) and *cbbL* (encoding the large subunit RuBisCo for autotrophic CO_2_ fixation). The *pmoA* gene is the most frequently applied phylogenetic marker to distinguish aerobic methanotrophs from other bacteria in different ecosystems (McDonald et al. 2008; Lau et al. 2013).

The genus *Methylobacter,* belonging to the family *Methylomonadaceae* (Type Ia), was initially proposed by Bowman and collaborators in 1993 (Bowman et al. 1993). Currently, the genus now contains 8 validly published species (Collins et al. 2015 and 2017). All members of the genus *Methylobacter* are strictly aerobic, rod-shaped, and capable of aerobically oxidizing methane to carbon dioxide. Moreover, cells assimilate carbon by using the ribulose monophosphate (RuMP) pathway and possess an arranged stack membrane system in its cell compartment (i.e. a Type I intracytoplasmic membrane). Until now, none of the reported species of the genus *Methylobacter* has been found to maintain soluble methane monooxygenases (sMMO) (Houghton et al. 2019). *Methylobacter* species generally produce pink, yellow, or white color colonies on solid medium and have been isolated from various environments like wetland soil, sediments, freshwater, and rumen (Wartiainen et al. 2006; Whittenbury et al. 1970; Finn et al. 2012; Khatri et al. 2021).

Here, we report on the microbial community’s comparative analyses and distribution patterns with emphasis on methane oxidizing-bacteria at two different sites close to the methane emission source in Lagoon Pingo. We used sediment samples from Lagoon Pingo, where methane is enriched, and oxygen-limited groundwater is discharged continuously, forming crater ponds (Hodson et al.^2019^). Initially, molecular community analyses at this site revealed a distinct and unusual methanotrophic community assemblages across hydrological transitions (Fåne 2020). In one of our enrichments, we recovered an extant cold-adapted, obligate psychrophilic bacterium, which was assigned LS7-T4A^T^. This isolate showed high 16S rRNA gene sequence similarity to members of the genus *Methylobacter* and low similarity to other methanotrophic genera in the family *Methylomonadaceae.* For further verification of the taxonomic position of this strain, a polyphasic characterisation and a genomic overview were implemented to give valid evidence of the novelty of this new isolate.

## Materials and methods

### Site description and sample collection

**Fig. 1:**
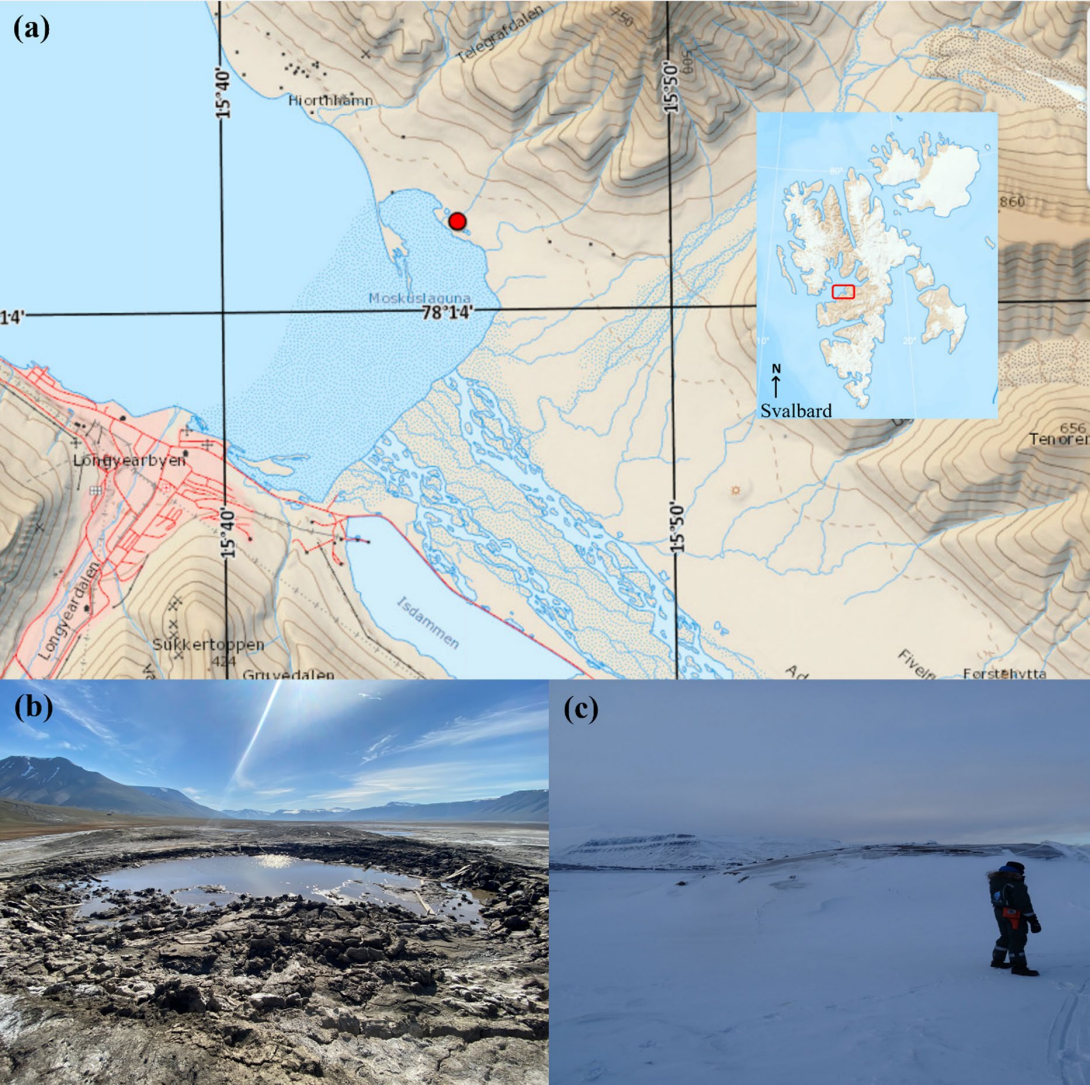
Sample site showing the location of Lagoon Pingo indicated with a red circle situated close to Moskuslagoon near Adventdfjorden in Svalbard at 78°N **a** shows the geographical location of Lagoon Pingo on map taken from toposvalbard **b** picture taken during summer 2021 showing the Shallow Lake with crater-like structure **c** image showing elevated Ice-hill structure covered in snow during winters

Sediment samples were collected from Lagoon Pingo in Adventdalen Valley, located on the Northern side of Adventdalen River close to Longyearbyen, Svalbard (78°14.403′N, 15°45.281′E) in early August 2019 (Fig. 1a). The pingo is separated from tidal waters by the Moskuslagoon near the coastline. The site is an open pingo system and collapses as a shallow crater lake during summer (Fig. 1b) and builds up as an icy hill during winter and spring (Fig. 1c). The system consists of several elevated mounds with craters spanning 500 m in length, 150 m in width, and up to 10 m in height (Yoshikawa and Nakamura 1996).

**Fig. 2:**
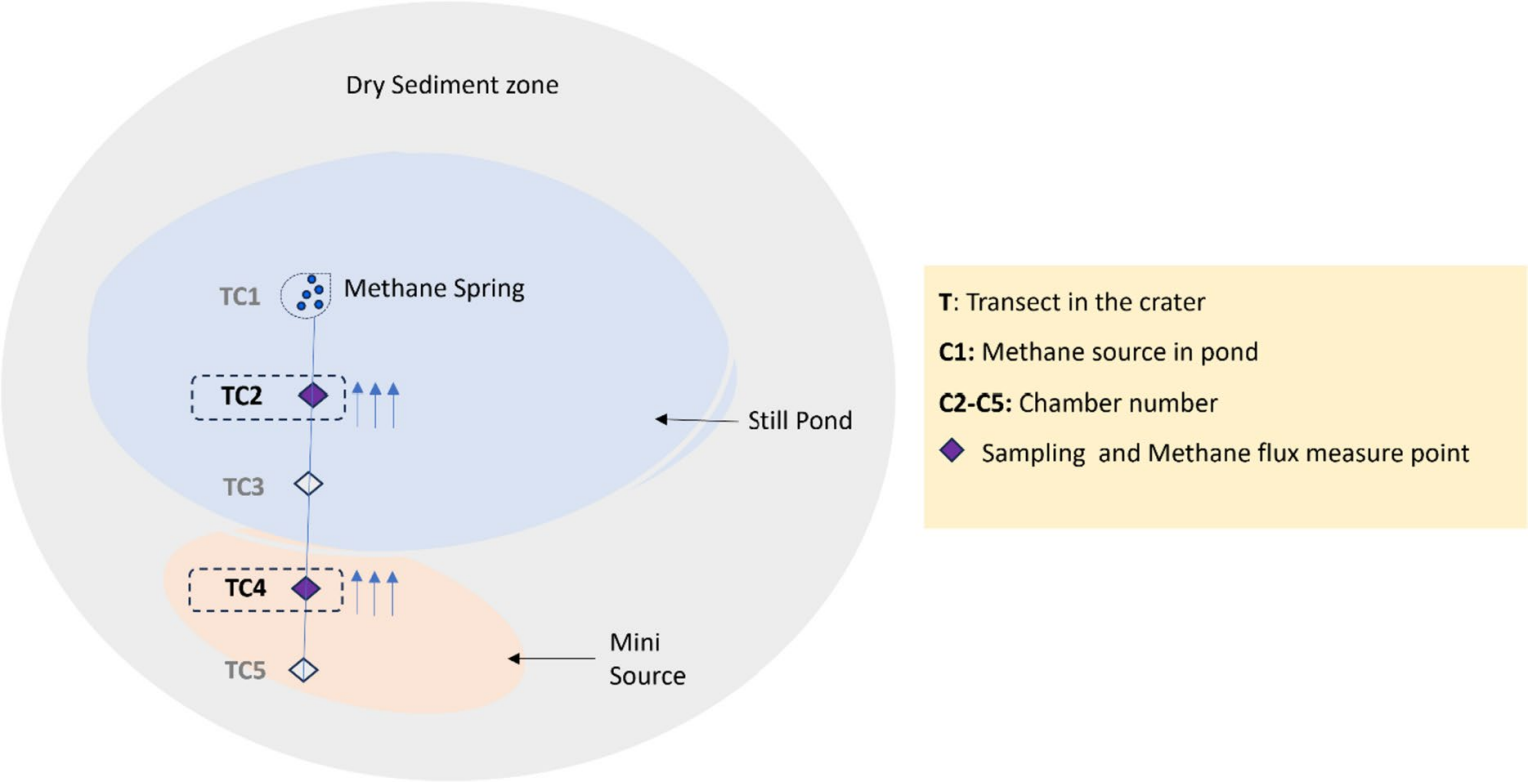
A schematic drawing of Lagoon Pingo showing the still pond (SP) in light blue and the mini source (MS) in light pink, surrounded by dry sediment indicated with the grey color. Samples from the transect T, which is indicated as a straight line with five methane measurement and sampling locations, was used in this study. The SP consists of a water-logged methane spring at TC1 with a high methane flux indicated by blue bubbles. The diamond shapes indicate where methane flux chambers were located, starting from the centre towards the dry sediment zone. At the locations of the methane flux chambers, sample collection was also performed (Fåne 2020). Samples from the locations of chambers TC2 and TC4, shown in violet diamond shapes, were included in the current study. The temperatures of the collected material at TC2 and TC4, were 10.0 and 5.4 °C, respectively

Lagoon Pingo has three active springs (Hodson et al. 2020). The samples used in this study were collected from the accessible dry sediment area at the rim of the active spring at Lagoon Pingo (Figs. 1b, 2). During autumn, when samples were collected, the structure of the site consisted of a still pond (SP) with methane seeps identified by ebullition to the surface. The main methane spring was situated in the centre, discharged water saturated with methane, and a temperature of 0.5 °C at the surface (Fig. 2).

The sediments used were collected from transect 2 (T) from the methane spring from the middle of the pond into the rim, where an additional “mini methane source” (MS) was located (shown in Fig. 2) resulting in five sampling points labelled as the chamber (C1–C5). Replicate samples were taken from TC2 closer to the main spring from the still pond (SP) with a temperature of 10 °C and from TC4 with small discharges called mini source (MS) where the temperature was 5 °C, at a depth of 10 cm. The recorded pH on the site was 9, which indicates an alkaline environment. Sediment samples collected for nucleic acid extraction were immediately frozen in a portable dry shipper (Air Liquide, Paris, France) on site (< − 150 °C). pH measurements were performed in 1:5 dilution (sediment:MilliQ water). Samples for enrichment were stored cold at 4 °C in a sterile serum vial with thick rubber septum. Samples were kept cold during transportation, until further processing in the laboratory at University of Bergen, Norway.

### Methane fluxes

Net methane fluxes were measured using custom-made, static acrylic glass chambers (3603 cm3 inner volume) in combination with a recirculating multiplexer (eosMX-P, Eosense, Dartmouth, Canada) and OA-ICOS ultraportable greenhouse gas analyser (U-GGA-915, Los Gatos Research, San José, USA). Inert, gas-tight perfluoro alkoxy alkanes polyurethane tubing was used as a gas line for the gas transfer between chambers, multiplexer, and greenhouse gas analyser. Before each methane flux measurement, the setup was flushed with ambient air. Depending on the magnitude of methane fluxes, measuring time amounted to 5 min. During each flux measurement, chamber temperature and pressure were monitored using a temperature logger (HOBO MX2201, Onset, Cape Cod, USA) and a manometer (Leo1, Keller AG, Winterthur; Switzerland). The net methane flux estimates were determined by linear regression implemented in the eosAnalyse-AC program (Version 3.7.9, Eosense, Dartmouth, Canada). The accuracy and consistency of the greenhouse gas analyser were periodically checked by referencing ambient air and a standard gas (1000 ppm methane in N2).

### DNA extraction, sequencing of 16S rRNA gene, and assignment of taxonomy

Environmental DNA (eDNA) was extracted using the DNeasy PowerSoil Kit (QIAGEN, 12,888–100, Germany) using manufacturers protocol. Extracted DNA was quantified using a high-sensitivity kit of Qubit 2.0 Fluorometer (Invitrogen, Singapore) following the manufacturer’s instructions and then stored at − 20 °C. The eDNA was amplified by targeting the highly conserved V4 -region of the 16S rRNA gene by using nested polymerase chain reaction (PCR) as previously described (Wilson et al. 2017). The 16S rRNA gene amplicon libraries were sequenced on an Illumina MiSeq platform (Norwegian Sequencing Centre, Oslo).

The demultiplexed pairedend fastq sequences were analysed using the DADA2 pipeline (Divisive Amplicon Denoising Algorithm 2, Callahan et al. 2016) using default parameters. Sequence qualities were verified using plotQualityProfile and low-quality reads were removed using filter and Trim function. Primers were removed using the cutadapt function. The core method from DADA2 packages was applied using multithreads to infer the composition of samples. Paired readwere merged to obtain full denoised sequences (merged sequences) using dereplication function. amplicon sequence variants (ASVs) table was made using Seqtab. Chimeric sequences were removed from merged reads using nochim function and taxonomy assigned to the ASVs using the assign taxonomic function in DADA2 package which is based on naïve Bayesian classifier method and SILVA reference database (Quast et al. 2012). ASVs showing bootstrap values above 90 were included in further analyses and subsamples were presented in the pie charts. The statistical analysis used the online MicrobiomeAnalyst platform (Dhariwal et al. 2017).

### Enrichment and isolation of aerobic methanotroph

To enrich for isolation of methanotrophic bacteria, 2 g sediment from mini source (MS) was selected and inoculated in 20 mL low-salt mineral media (both LMM: added vitamin solution and LMA: without vitamin solution) in 120 mL serum vial closed with a sterile butyl rubber septum and sealed with aluminium crimps. The pH was adjusted to 8.5, and the substrate for growth was a sterile mixture of methane (80%) and air (20%) in the headspace (purity of methane, 99.5%, Yara Praxair, Oslo, Norway) as previously described for LMM and LMA medium (Islam et al. 2015; Islam et al. 2016). The bottle was incubated at 10 °C for 4 weeks in the dark, without shaking. The gas mixture was substituted every 15 days. When the enrichment culture became visibly turbid, they were checked for cell growth using phase-contrast microscopy (Eclipse E400 microscope, Nikon Corporation, Tokyo, Japan). Two mL of primary enrichment cultures were transferred to fresh LMM and LMA media and re-incubated under the same conditions. To recover a factual aerobic methane oxidiser, the enriched sample was transferred five times in fresh media and incubated with a combination of methane and air. Serial dilutions (10^−6^ to 10^−8^) were prepared, and 0.1 mL aliquots were spread onto agar plates (Difco) containing LMA medium. The plates were incubated for 5 weeks at 15 °C in gas-tight jars filled with methane gas and air in a 2:1 mixture. One single colony was then selected, re-streaked onto fresh agar plates, and re-incubated for 5 weeks. After the pure culture was obtained, LMA was used for its routine cultivation at 10 and 15 °C at pH 8.0 for 2 weeks. The purity of the cell culture was again checked by phase-contrast microscopy. A heterotrophic contamination test used glucose (10 mM), yeast extract (5%), and R2A agar plates.

### Phylogenetic classification

The cells of strain LS7-T4A^T^ were targeted for the amplification of genes, including 16S rRNA genes, *pmoA, mmoX, mxaF, nifH, cbbL* and *mauA* using specific primers (list of primers given in Table S1) and positive amplification products sequenced as described previously (Islam et al. 2020)0.16S rRNA gene sequences and protein sequences of the *pmoA* gene inferred from PCR products using the ExPASy Translate tool (Artimo et al. 2012) were compared to available sequences from the GenBank database using the NCBI tools of Blastn and Blastp, respectively. Phylogenetic trees of both 16S rRNA and *pmoA* genes were reconstructed using the neighbor-joining (NJ) and maximum likelihood (ML) in MEGA software version 7.0 (Kumar et al. 2016).

### Physiology and TEM characterization

Different organic substrates (glucose, acetate, pyruvate, lactate, malate, succinate, and ethanol) were tested at concentration of 10 mM in fresh LMA (Islam et al. 2008). Growth on methanol, methylamine, formate, and formaldehyde were examined at concentrations from 0.03 to 0.2% (v/v) in an LMA medium. Moreover, growth was tested with nitrogen-free LMA (without KNO_3_ or NH_4_Cl) adjusted to pH 8.0 in triplicates, where the only nitrogen source was N_2_ from the air. The samples were monitored during the incubation time and observed every week for visible growth. After 2 weeks of incubation, growth could be observed. Salt tolerance was determined by adding different concentrations of NaCl (0.1, 0.5, 1.0, 2.0, and 3.0% w/v) to the LMA medium. After 2 weeks of incubation the turbidity of each sample was assessed at 600 nm using a spectrophotometer. The generation time and the growth rate (µ) at 15 °C and pH 8.0 on methane were determined from the exponential growth phase. Growth measurements were recorded after 2 weeks of incubation. To determine optimum temperature for growth, the culture was incubated at 0, 2, 5, 8, 10, 13, 15, 18, 20, 22, 25 and 30 °C (at pH 8.0) with methane as the only available carbon source. The influences of pH on growth were recorded, and antibiotic sensitivity of strain LS7-T4A^T^ was examined at the optimum temperature of 15 °C and pH 8.0 as previously described (Islam et al. 2020). The morphology was studied using phase-contrast microscope, and the internal structures of pure cells were evaluated using transmission electron microscopy (TEM, Hitachi HT7800) as described by Islam et al. (2015).

### Metagenome sequencing, assembly, and annotation

Total genomic DNA was extracted from strain LS7-T4A^T^ using GenElute Genomic DNA kit (Sigma), and the metagenome was sequenced using short-read Illumina sequencing platform (Illumina Novoseq 6000 platform: Novogen Co. Ltd., Cambridge, UK). Library preparation, sequencing for short read and annotation was done at Novogene Co. Ltd. To ensure the accuracy and reliability of the subsequent information analysis results, the original data were filtered by the step of quality control using the Novogen compiling pipeline. The genome was assembled using defalt K-mer of three different softwares (1) SOAP denovo version 2.04 (Li et al. 2010) (2) SPAdes (Bankevich et al. 2012a, b) (3) Abyss (Simpson et al. 2009). The assembly results of the three softwares were integrated with CISA software (Lin and Liao 2013) and the assembly result with the least scaffolds was selected. The genome was subjected for prediction of the coding gene using GeneMarkS (Besemer et al. 2001) Transfer RNA (tRNA) genes were predicted by tRNAscan-SE (Lowe et al. 1997). Ribosomal RNA (rRNA) genes were analyzed by the rRNAmmer (Lagesen et al. 2007). Small nuclear RNAs (snRNA) were predicted by Rfam (Gardner et al. 2009). Among several database for gene prediction KEGG is Kyoto Encyclopedia of Genes and Genomes (Kanehisa et al. 2004) and COG Clusters of Orthologous Groups) were used for functional annotation and investigation of the metabolic potential.

### Metagenome analyses, genome identity, and phylogeny

The phylogenomic tree was constructed based on the 16 whole genomes from the Methylomonadacecae family, which was created using the automated codon tree method in V-BRC Patric using protein homology groups and coding DNA from single-copy genes (Wattam et al. 2014; Davis et al. 2020). The genome identity analysis was done using ANI, AAI, and GGDC. The average nucleotide identity (ANI) values amid strain LS7-T4A^T^ and other associated species in the genus *Methylobacter* were calculated using JSpeciesWS (Richter et al. 2016) which is a web server for prokaryotic species circumscription based on pairwise genome comparison (Richter et al. 2016). Additionally, digital DNA-DNA hybridization (dDDH) values between strain LS7-T4A^T^ and other related species in the genus *Methylobacter* were acquired using the Genome-to-Genome Distance Calculator (GGDC) (Auch et al. 2010) using the method described by Meier-Kolthoff et al. (2013).

### Culture deposition and nucleotide sequence submission

The GenBank accession numbers for the sequences of the16S rRNA genes of strain LS7-T4A^T^ is OQ832782. The raw reads of 16S rRNA amplicon Illumina sequence data submitted in sequence read archive (SRA) accession numbers BioProject ID PRJNA1024519 in GenBank and draft genome sequence under the BioProject ID: PRJNA1024098.

## Results

### Microbial community diversity

**Fig. 3:**
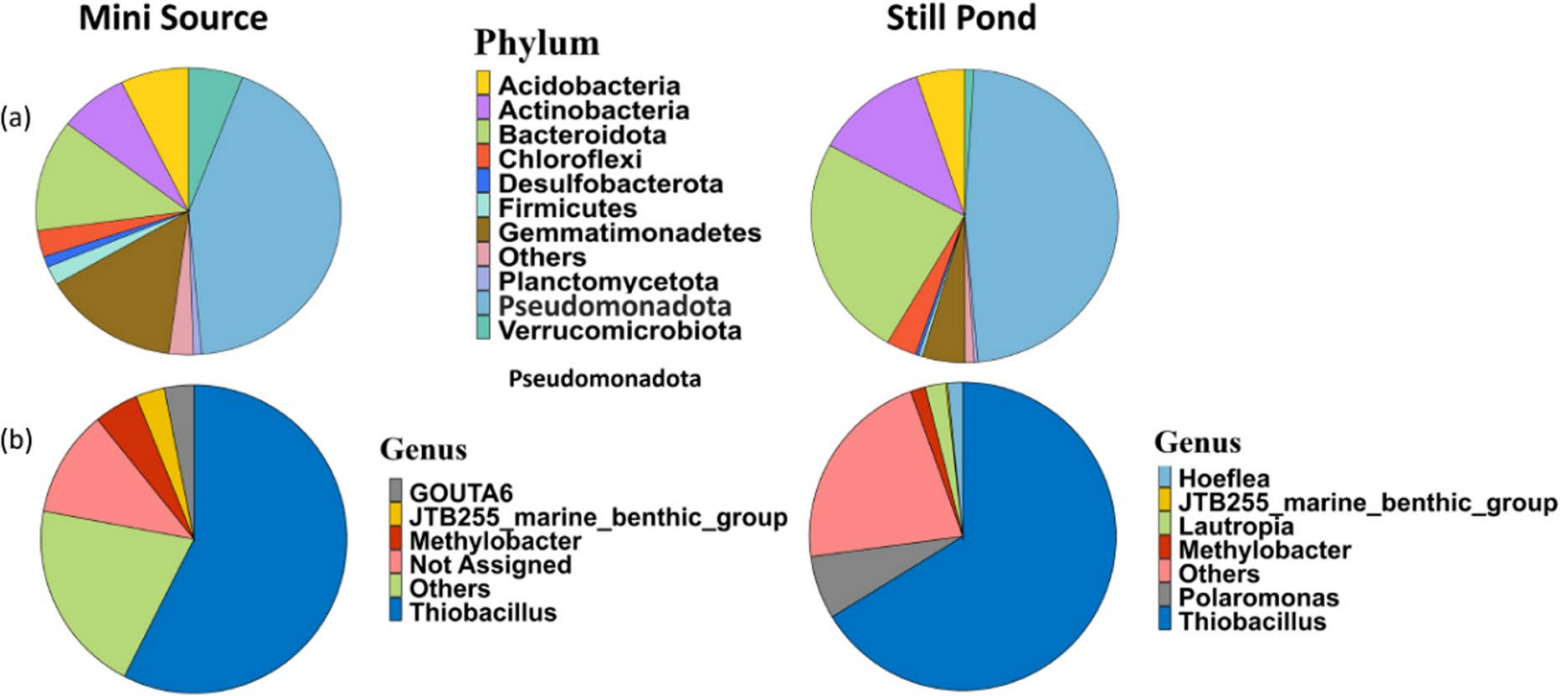
Microbial community composition in the two sediments samples mini source (MS) and still pond (SP) from Lagoon Pingo based on high throughput metabarcoding and Illumina sequencing. The pie charts show relative abundance at **a** the phylum level and **b** at the genus level, selectively showing distribution within the phylum *Pseudomonadota*

Samples from two locations in Lagoon Pingo, the mini source (MS) and still pond (SP), were used in this study. Each location was characterized by proximity to Lagoon Pingo’s central subsurface water discharge. Both locations were close to the primary water source (MS, 7.61 m and SP, 3.71 m) with differences in temperature (MS, 8.4 °C and SP, 11.4 °C) and methane fluxes (MS, 687.5 and SP, 1.5 nmol m^2^ s^−1^), but not water content (both waterlogged). The 16S rRNA genes were amplified and sequenced in DNA samples from four replicates for each location. A total of 2018 bacterial ASV (Amplicon Sequence Variants) were identified in the eight samples. All assigned ASVs belonged to the bacterial domain. The microbial communities were dominated by *Pseudomonadota* phylum with 47% and 42%, followed by *Bacteroidota* at 24% and 12%, *Actinobacteri*ota with 11% and 7%, *Acidobacteriota* with 5% and 7% followed by *Gemmatimonadota* 4% and 14% relative abundance in SP and MS, respectively, as shown in Fig. 3a. The MS source was dominated by the families of *Pseudomonadota* phylum *Burkholderiaceae* followed by *Hydrogenophilaceae* and the still pond was dominated by *Hydrogenophilaceae* followed by *Flavobacteriaceae* (*Bacteroidota* phylum) (Fig. S1). Further resolution of *Pseudomonadota* phylum showed the dominance of *Gammaproteobacteria*, composed of the three common genera *Thiobacillus* (57.5% and 66.0%), *Methylobacter* (4.7% and 1.6%), and JTB255_marine_benthic_group (3.0% and 0.9%) in MS and SP respectively Fig. 3b.

### Enrichment, isolation, and classification

**Fig. 4:**
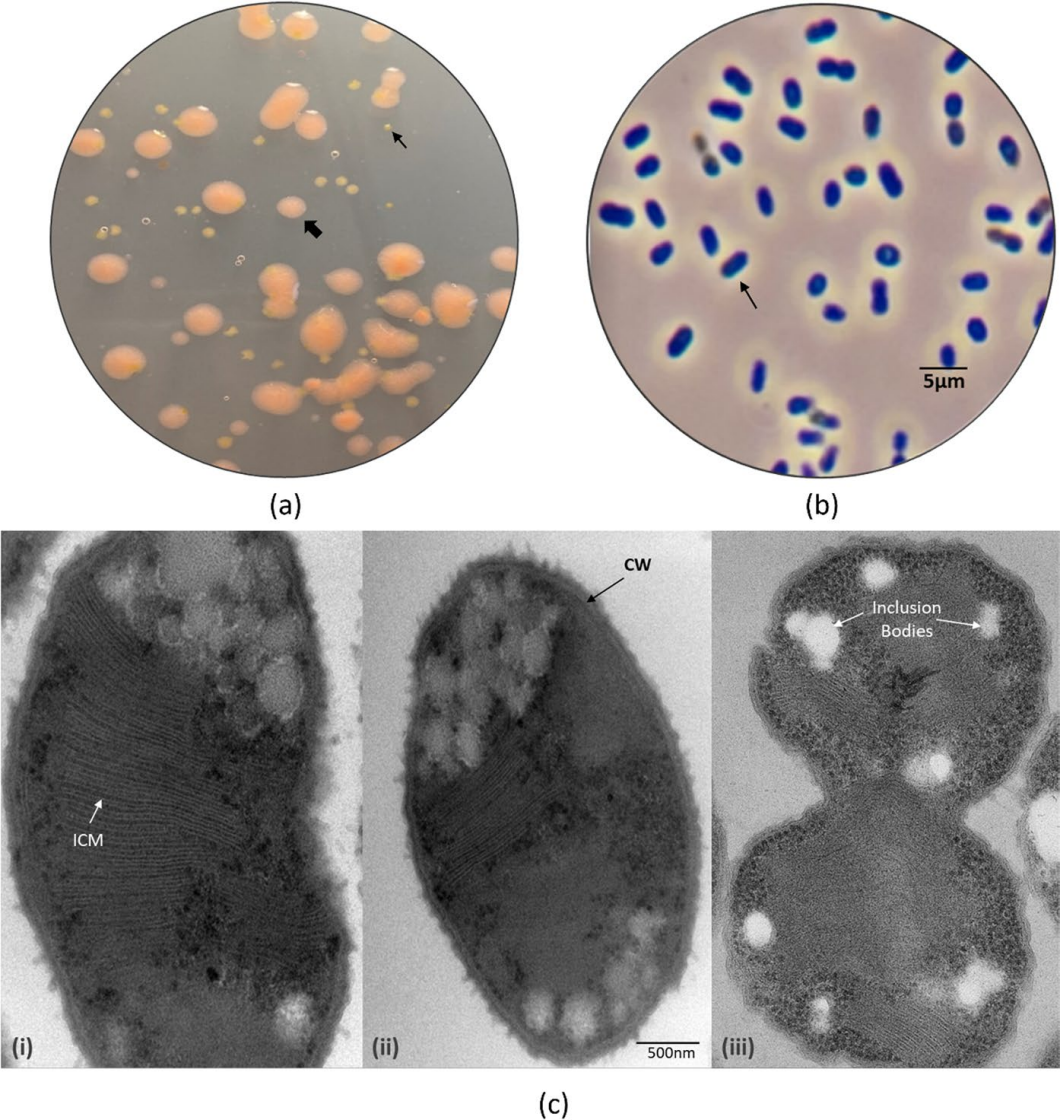
Morphological characteristics of the strain LS7-T4A^T^ **a** image of LMA agar plate showing light pink colonies Indicated with large arrow heads) and small white colonies (indicated with small arrow heads). The light pink colonies were examined under microscope and micrographs shown in **b** and phase- contrast micrograph of live cells showed rod-shaped bacteria. **c** Electron micrograph of a cross-section showing the internal characteristic of the cells (i– iii). White arrow (i) indicates the intracytoplasmic membranes (ICM) arranged in stacks, black arrow (ii) shows the cell wall (CW) and white arrow (iii) point to inclusion bodies observed as tiny white sacs under the microscope. Scale bar is 500 nm

Using sediment samples from the mini source, the enriched cultures grew after 5 weeks of incubation at 10 °C with methane as the only carbon source in LMA medium. Cells observed under phase contrast microscope were dominated by short rod-shaped cells with mucus-like capsules, with few coccoid cells and small thin rod cells after four consecutive transfers. Two distinct types of colonies were found after plating the enrichments on LMA agar plates. One of the colony types consisted of small white colonies about 0.4–0.6 mm in diameter; the other colonies were light pink coloured colonies about 1.8–2.5 mm in diameter. Under the microscope, the white colonies consisted of small rod-shaped cells, and the pink colonies were a mixture of coccoid and rod-shaped cells. Only the pink colonies sustained growth on methane after 5 weeks of incubation (Fig. 4). The pink colonies were used for further physiological and phylogenetic characterisation. The isolate was designated LS7-T4A^T^, which grew on methane or methanol as the sole carbon and energy source. No growth was found on multi-carbon substrates like glucose, acetate, succinate, malate, lactate, pyruvate ethanol, yeast extract, or R2A agar plates. The growth on different substrates confirmed that the strain was an obligate aerobic MOB that could grow on methanol. The purity of strain LS7-T4A^T^ was confirmed by microscopy in addition to 16S rRNA gene sequencing.

**Fig. 5:**
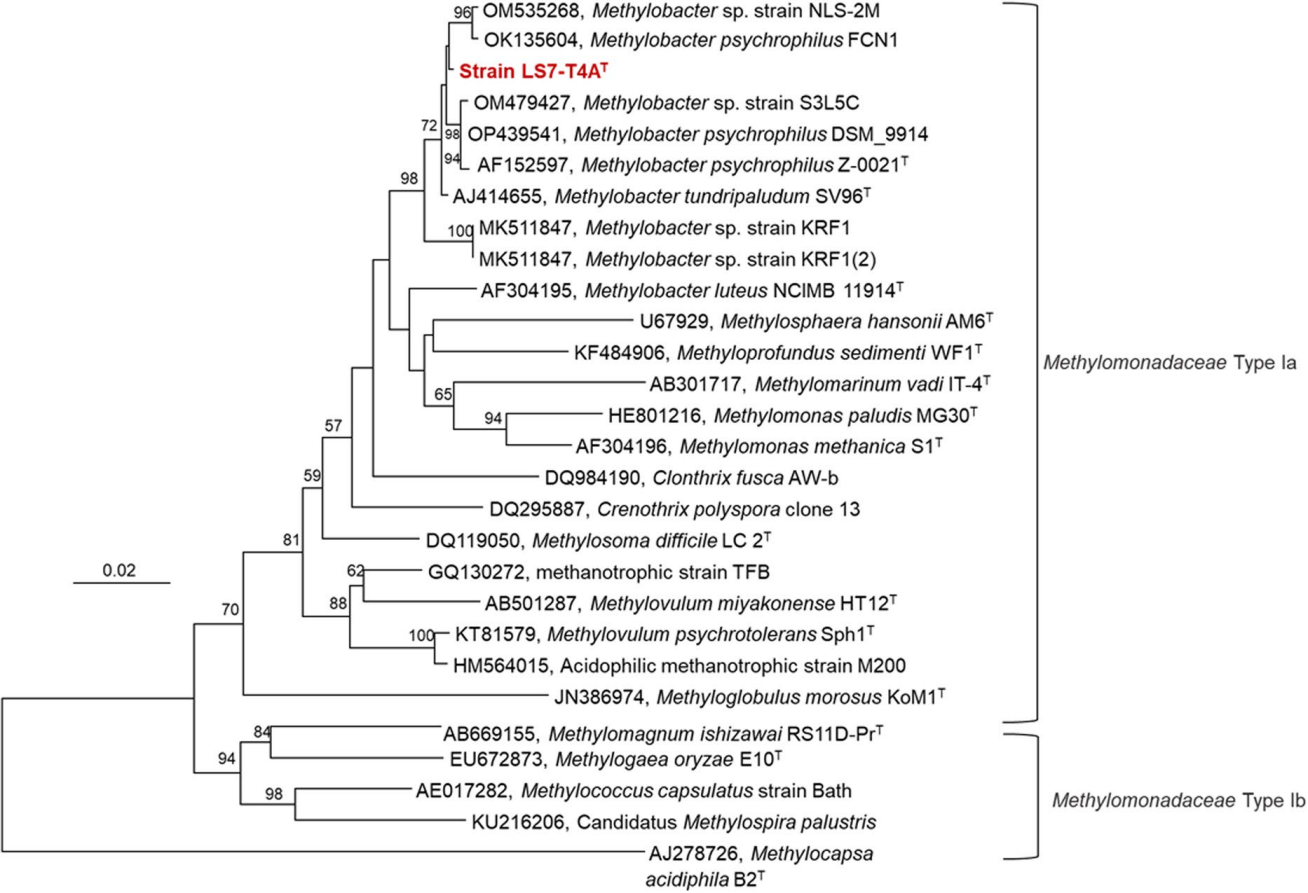
Neighbour-Joining (NJ) phylogenetic tree of strain LS7-T4A^T^ from Lagoon Pingo (showed in bold red) based on the analyses of the 16S rRNA gene using the Kimura 2-parameter model showing phylogenetic relationship related to strains from genus *Methylobacter* and other cultured and uncultured Type Ia and Type Ib genera from *Methylomonadaceae* and *Methylococcaceae* family. The Type IIb methanotroph, *Methylocapsa acidiphila* B2^T^ (AJ278726) of the family *Beijerinkiaceae*, was used as an outgroup. The number displayed at the branches refer to NJ-bootstrap values

**Fig. 6:**
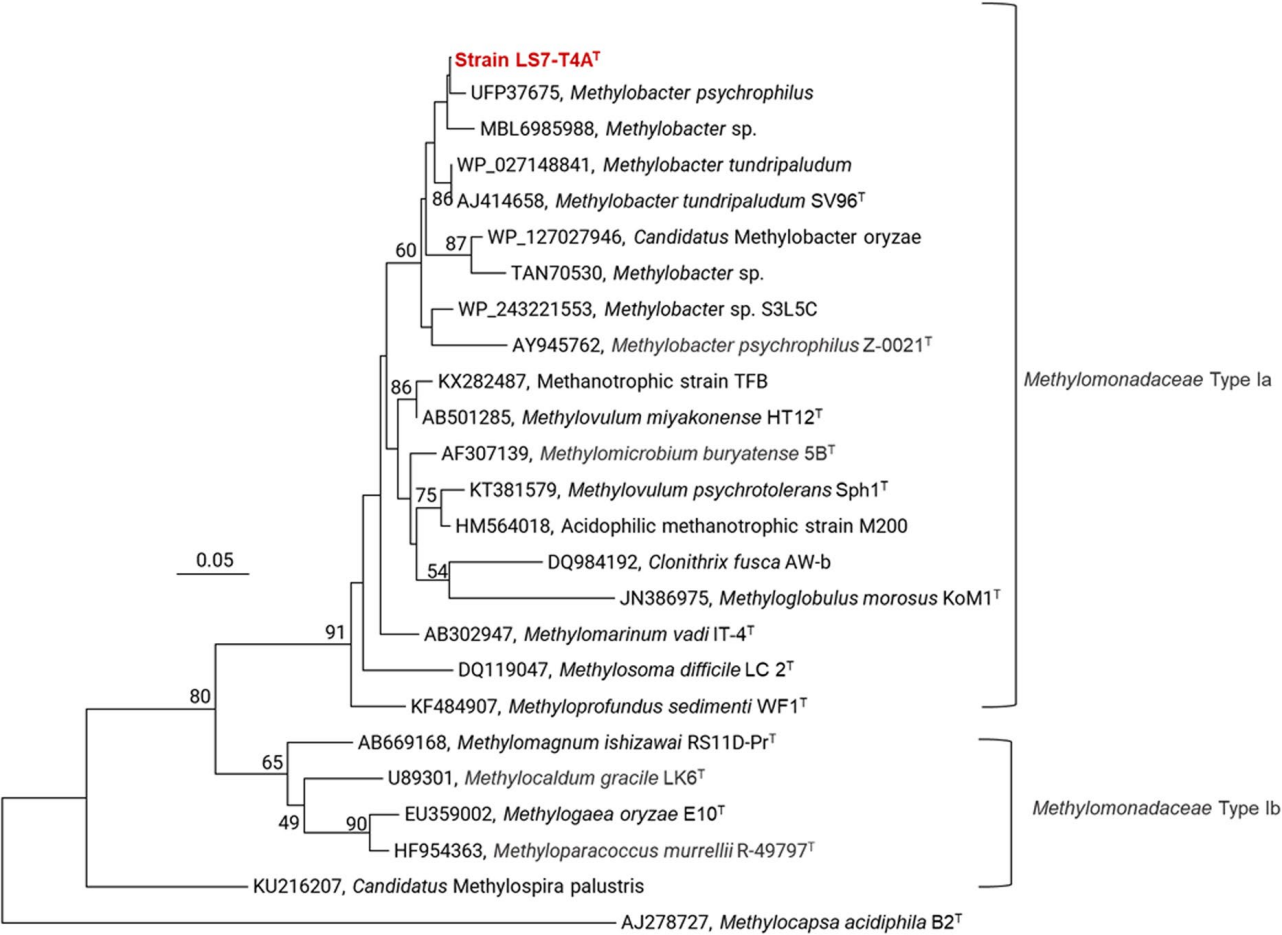
Neighbour-joining (NJ) phylogenetic tree derived using the pmoA gene amino acid sequences, based on Dayhoff matrix-based model showing the position of the strain LS7-T4A (showed in bold red) and other related Type Ia and Type Ib methanotrophs. The Type IIb methanotroph, *Methylocapsa acidiphila* B2^T^ (AJ278727) of the family *Beijerinkiaceae*, was used as an outgroup. The number displayed at the branches refers to NJ-bootstrap values

Phylogenetic analysis revealed that the 16S rRNA genes sequence cluster within the genus *Methylobacter* and the nearest cultivated species is Methylobacter tundripaludum SV96^T^, with a sequence identity at 99.06%. The second closest match was *Methylobacter* S3L5C with a sequence identity of 99.00% (Fig. 5). The *pmoA* gene (particulate methane monooxygenase subunit A), used as a biomarker gene for defining methanotrophic bacteria, was aligned and based on their gene sequences a phylogenetic tree was constructed (Fig. 6).

### Physiological and TEM features

The LS7-T4A^T^ isolate had an optimum growth temperature of 13 °C at pH 8.0 (Table S2). The growth rate declined after 13 °C, and no growth was observed at 25 °C (Fig. S2). The optimal pH was pH 8.0, and no growth was recorded at pH 6.0 and 9.5. Growth was inhibited when NaCl concentrations exceeded 0.5% (w/v). Growth was not achieved under aerobic conditions in the absence of methane or under anaerobic conditions in the presence of methane. Multi-carbon substrates prevented the growth of strain LS7-T4A^T^. Moreover, the strain was able to grow with low methanol concentrations, between 0.05 and 0.5%. The generation time and growth rate (µ) when growing on methane was 19 h and 0.016 h^−1^, respectively. All antibiotics tested inhibited the growth of LS7-T4A^T^. Ammonia and nitrate compounds were used by cells as nitrogen sources. Vitamins were not found to be required for growth. The strain was able to grow on nitrogen-free LMA and LMM, indicating the ability to fix atmospheric N_2_. Still optimal growth was observed on LMA (containing NH_4_Cl) compared to LMM (containing KNO3). These observations were also supported by positive amplification of the *nifH* gene (Fig. S3). The strain was non-motile, and the cells multiply by binary fission. No flagella were visible by transmission electron microscopy. The ultrathin sections in TEM analysis showed the presence of extensive intracytoplasmic membranes, close-packed in vesicular disks (as stacked), which is a typical feature of the family *Methylomonadaceae* (Fig. 4c)*.*

### Genome features of methylobacter strain LS7-T4A^T^

To better understand the genome feature and metabolism of strain LS7-T4A^T^ the genome was sequenced, and draft genome assembled. The metagenome-assembled genome was constructed using 220 contigs giving a total size of 4,338,157 bp. The genome indicated 99.7% completeness with only 0.845% contamination. The GC content was 47.93%, and the genome included one rRNA operon, 41 tRNAs, and 4271 total number of genes. Genome features are summarized in Table 1.

**Table 1:** Genome Statistics of *Methylobacter* LS7-T4A^T^

Attribute	Value
Genome size (bp)	4,316,197
DNA scaffolds (> 500 bp)	220
DNA G+C content (%)	47.92
N50 length (bp)	65,853
Number of tRNA	41
Number of rRNAs (5S, 16S and 23S)	3
Total gene number	4,271
Gene total length (bp)	3,626,817
Gene length/Genome (%)	84.15
Gene average length (bp)	849
Genes assigned to COGs	2,888
Proteins families (Pfam)	263
KEGG function-specific prediction	3474

bp, basepairs

**Fig. 7:**
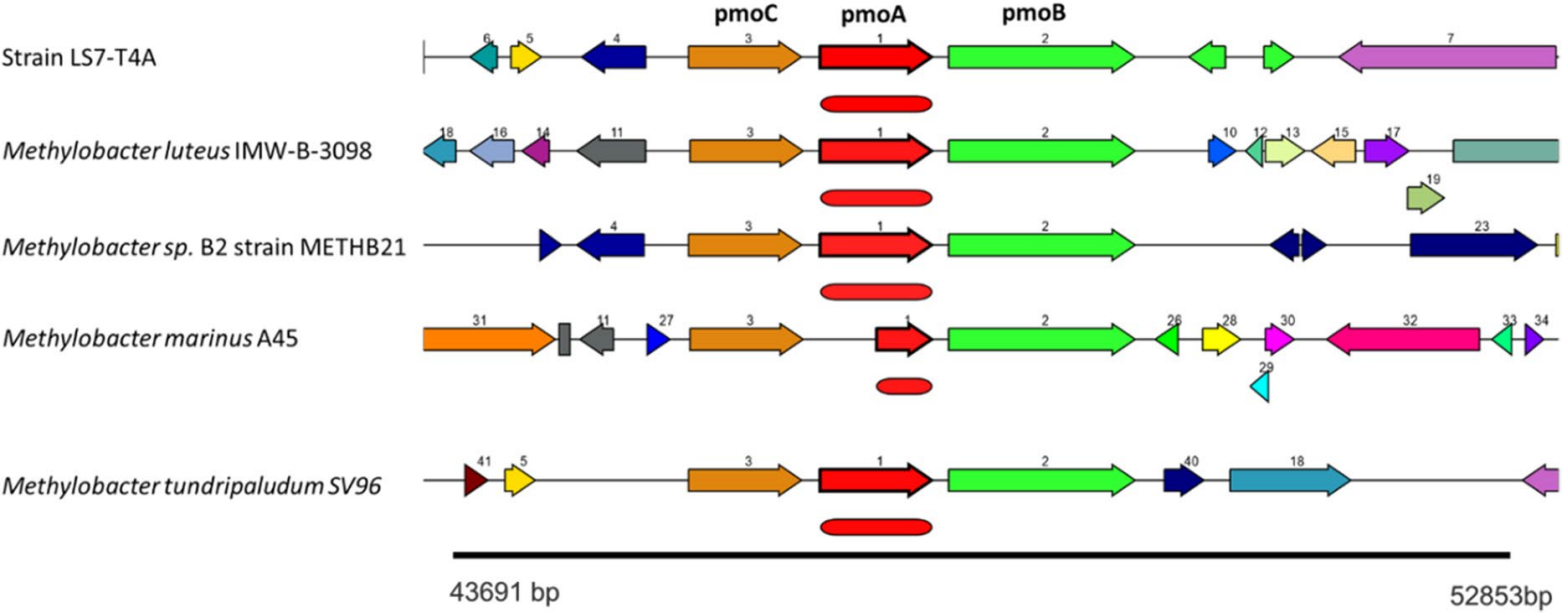
Gene organization of particulate methane monooxygenase gene *pmo*CAB in strain LS7-T4A^T^ compared to four other species of the genus *Methylobacter*

Of the predicted genes, 84.85% were assigned to Clusters of Orthologous Groups (COGs). The genome of strain LS7-T4A^T^ had one complete sequence of 16S rRNA gene sequence, with 1530 bp on scaffold no.112 and the *pmoA* gene (particulate methane monooxygenase subunit A), was complete 744 bp) and present in the genome on scaffold no 17 (Fig. 7).

The average nucleotide identity (ANI) values were determined using online resources JspeciesWS (Richter et al. 2016) were 85.51% with *M. tundripaludum SV96*^*T*^ and 75.63% *Methylobacter* S3L5C. The dDDH values were 31.70% with *M. tundripaludum SV96*^*T*^ and 22.40% *Methylobacter* S3L5C (Table 2). The percent identity of the sequence was 97.57% with *M. tundripaludum SV96*^*T*^ and 97.98% with *M. psychrophilus.* ANI values below 95–96% for Bacteria and Archaea can be considered as a novel species (Yoon et al. 2017).

**Table 2:** Pairwise comparison of average nucleotide identity (ANI) values of *Methylobacter* isolate LS7T4A, compared with other species of the genus *Methylobacter*

No	Strain name	1	2	3	4	5	6	7	8	9	10	11
1	*Methylobacter*LS7-T4A^T^	*	75.54	75.88	75.63	80.07	74.40	85.24	85.35	74.82	74.46	85.51
2	*Candidatus Methylobacter favarea* METHB21	75.77	*	74.19	74.16	74.12	73.79	75.88	75.89	74.16	73.72	75.86
3	*Methylobacter psychrophilus*	75.71	74.05	*	92.52	73.44	71.79	75.18	75.16	72.22	71.86	75.48
4	*Methylobacter* sp.S 3L5C	75.84	73.84	92.65	*	73.79	71.67	75.29	75.43	72.32	71.81	75.50
5	Candidatus *Methylobacter oryzae* KRF1 C707	79.78	73.76	73.23	73.33	*	73.25	81.21	81.21	73.75	73.30	81.23
6	*Methylobacter* sp.* BBA5.1*	74.20	73.32	71.90	71.70	73.35	*	74.37	74.43	84.14	98.47	74.39
7	*Methylobacter tundripaludum* 31/32	84.85	75.45	75.22	75.03	81.37	74.31	*	97.89	74.85	74.31	95.33
8	*Methylobacter tundripaludum* 21/22>	85.07	75.46	75.17	75.07	81.52	74.54	98.09	*	75.03	74.49	95.55
9	*Methylobacter luteus*IMV-B-3098>	74.59	73.81	72.33	72.18	74.03	84.16	74.93	75.03	*	84.31	74.95
10	*Methylobacter marinusM5*	74.33	73.32	72.01	71.95	73.58	98.61	74.43	74.57	84.31	*	74.57
11	*Methylobacter tundripaludum SV96*^*T*^	85.27	75.45	75.47	75.11	81.53	74.44	95.36	95.55	74.79	74.41	*

**Fig. 8:**
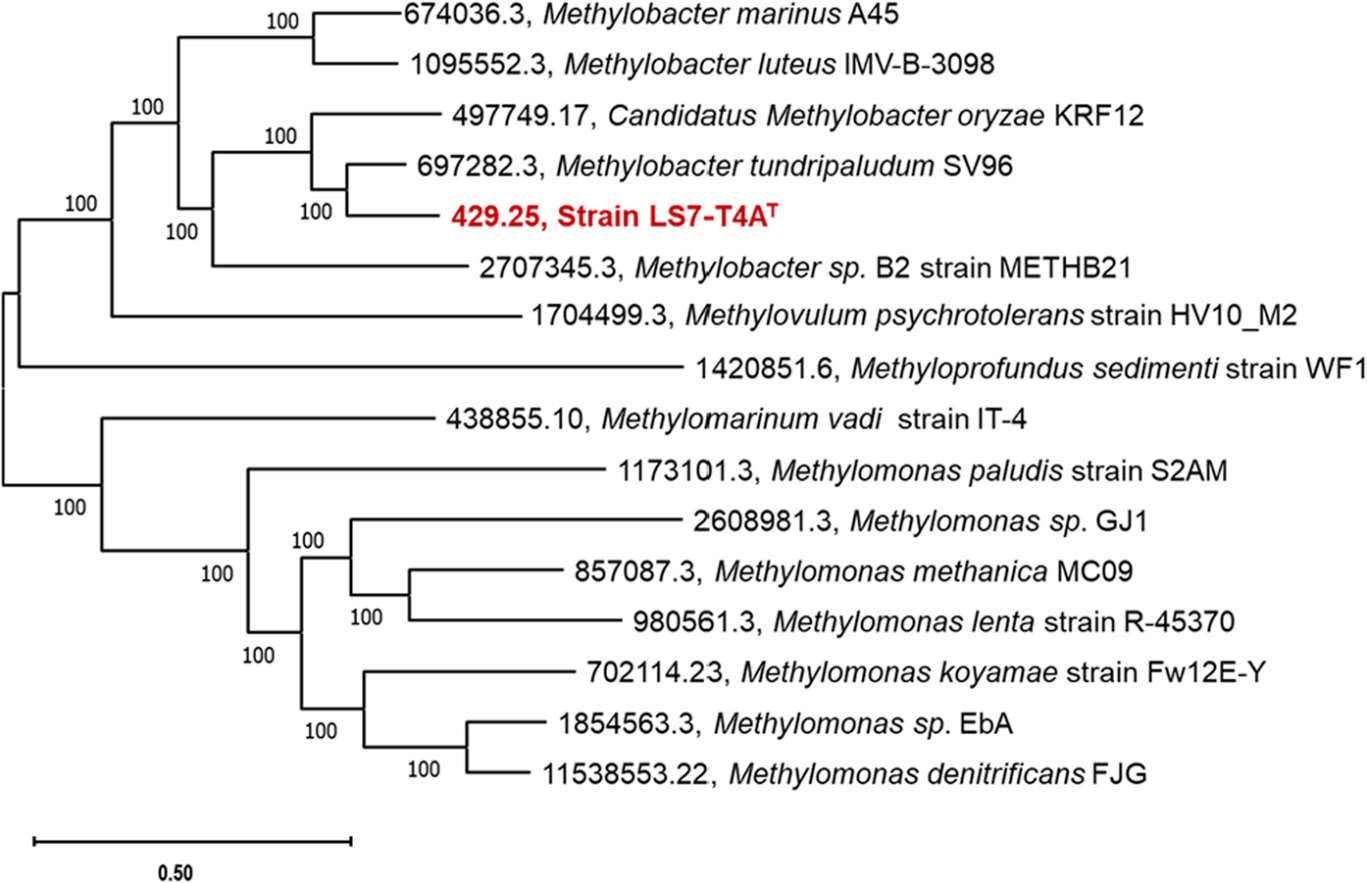
Phylogenetic tree of strains from Methylomonadacacae family relative to isolated strain LS7-T4A^T^ (in bold red) constructed by the Codon Tree method used on BV-BRC online service. The tree was based on the total number of 16 genomes, 100 aligned proteins, 45,521 amino acids and 136,563 aligned nucleotides and 100 CDS from the genomes

To validate the novelty of the LS7-T4A^T^ strain in the genus *Methylobacter*, the genomic tree with reported *Methylobacter* strains was constructed using BV-BRC Patric phylogenomic function (Davis et al. 2020). The phylogenomic analysis of the LS7-T4A^T^ showed that strain *M. tundripaludum SV96*^*T*^ and *Ca. Methylobacter oryzae* were the closest relatives, followed by strain *Methylobacter* S3L5C (Fig. 8).

## Predicted metabolic potential

### Methanotrophy

**Fig.9:**
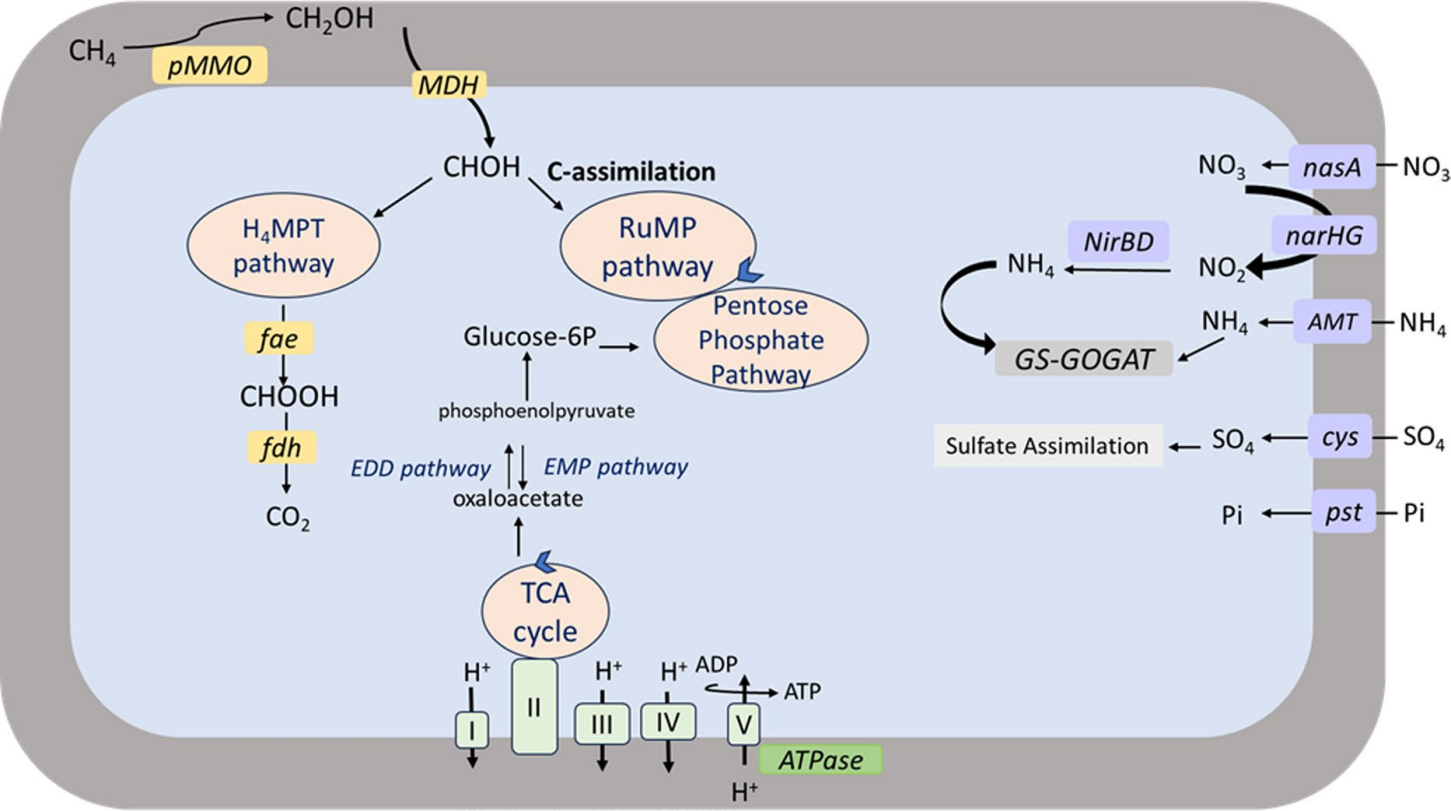
Cellular putative metabolic pathways reconstructed from the genome of *Methylobacter* LS7-T4A^T^. The carbon pathways are shown in circles and the genes involved in methane oxidation are shown in yellow boxes pMMO (particulate methane monooxygenase), MDH (methanol dehydrogenase), *fae* (5,6,7,8-tetrahydromethanopterin hydro-lyase), *fdh* (formate dehydrogenase). The genes shown in the green boxes represents electron transport chain complexes (I, II, III, IV, V). The cellular transporter genes on the right side in purple boxes are involved in nitrate assimilation and GS-GOGAT ammonia assimilation pathway *nas*A (assimilatory nitrate reductase), *nar*HG (dissimilatory nitrate reductase), *nir*BD (nitrite reductase), AMT (ammonia transporter), GS-GOGAT (glutamine synthetase-glutamine2-oxoglutarate aminotransferase). Followed by the sulfur assimilating gene *cys* (cysteine synthase) and *pst* (inorganic phosphate transport genes) into the cell

The draft genome analysis of strain *Methylobacter* LS7-T4A^T^ revealed that genes for *pmoCAB* of the particulate membrane-bound methane monooxygenase (pMMO), the first step of converting methane to methanol were detected. We did, however, not detect the soluble methane monooxygenase (sMMO) coding gene clusters. The enzymes in subsequent steps of the CH_4_ oxidation pathways transforming methanol to formaldehyde were also revealed (Fig. 9). The genome contained genes encoding subunits of the Ca-dependent methanol dehydrogenase (*mxaFI*) but lacked genes encoding the lanthanide-containing pyrroloquinoline quinone (PQQ) dependent methanol dehydrogenase (*xoxF*).

Screening the genome revealed the presence of genes encoding the enzymes for a complete tetrahydromethanopterin (H_4_MPT) C-transfer pathway for formaldehyde oxidation to formate. This included genes for the formaldehyde-activating enzyme (*fae*), the NAD(P)-dependent methylene tetrahydromethanopterine dehydrogenase (*mtdB*), methenyl-H_4_MPT cyclohydrolase (*mch*), and formylmethanofuran dehydrogenase subunits BCA (*fwd*BCA). We also detected the genes encoding the major subunit (*fdh*F and *fdo*G) and delta subunit (*fds*D) of format dehydrogenase, responsible for oxidation formate to CO_2_.

### Carbon fixation

The strain LS7-T4A^T^ contained a complete set of genes for the ribulose monophosphate pathway for carbon fixation from formaldehyde. Hexose phosphates are initial products formed by the condensation of formaldehyde and ribulose-5-phosphate. The key enzymes of the RuMP pathway are hexose phosphate synthase, encoded by *hxl*A, and phosphohexulose isomerase, encoded by *hxl*B which both were found in the genome of strain LS7-T4A^T^. The strain will thus likely assimilate carbon through RuMP pathway as shown in Fig. 9. The genome lacked key enzyme serine-glyoxylate aminotransferase encoded by gene *sga* for serine pathway and genes encoding the ribulose- 1,5-bisphosphate carboxylase/oxygenase /RuBisCO).

### Energy conservation and respiration

The strain LS7-T4A^T^ is obligate aerobe and uses O_2_ as a terminal electron acceptor. Energy conservation is through oxidative phosphorylation. the respiratory complexes comprised of nadh-quinone oxidoreductase (electron transport chain (ETC) complex I, characterised by the genes *nuo BCDHJKNM* were found, in addition to succinate dehydrogenase (ETC complex II, with the gene *sdh*) and cytochrome c oxidase (ETC complex III with the genes *cox*A and *cox*B) followed by F-type ATPase (ETC complex V, genes *atp*ABCDEFGHIK) as shown in Fig. 9. This validates a complete aerobic respiration chain present in our isolate.

### Nitrogen, sulfate, and phosphate metabolism

Genome analyses of the LS7-T4A^T^ strain indicated that it has the potential for using ammonia as a nitrogen source. The genes for the membrane bound ammonium transporter (AMT) was found in addition to the genes *gln*A (glutamine synthetase) and GDH2 (glutamate dehydrogenase), demonstrating its assimilation of ammonia through the glutamin synthetase/ glutamate synthetase (GS/GOGAT) system and providing available nitrogen for cellular anabolism. Potential for nitrate and nitrite assimilation was found by genes encoding ABC-type nitrate transporter (*Nas*A) together with the membrane bound nitrate reductase (and *Nar*HG, large and small subunits) and conversion of nitrite to ammonium by assimilatory nitrite reductase (*Nir*BD). The molecular marker gene for denitrification *Nir*K was not found. The strain also possesses genes for the nitrogen fixation process. Nitrogenases genes nifHDK (alpha and beta chain), *nif*E (co-factor synthase protein), *nif*N (iron protein), and *nif*W (nitrogenase-stabilizing/protective protein) were found. These include sulfate adenylyltransferase subunit 2 (*cys*D), bifunctional enzyme (CysN/CysC), phosphoadenosine phosphosulfate reductase (*cyc*H), sulfite reductase (NADPH) hemoprotein beta-component (*cyc*I), and tRNA 2-thiouridine synthesizing protein A (*Sir*). For dissimilatory sulfate reduction and oxidation, encoded by tRNA 2-thiouridine synthesizing protein (*Dsr*HCEF) was present but lacked specific genes like *sat Apr*AB and *Dsr*AB.

The genome showed potential for an inorganic phosphate transport system (*PstI)* to incorporate inorganic phosphate. This transport system is comprised of a periplasmic substrate-binding protein (*pst*S), a membrane-bound protein (*pst*A and *pst*C), and a protein that releases free Pi in the cytoplasm (*pst*B).

## Discussion

Several pingos situated along the Adventdalen Valley in Svalbard are formed by a combination of climate, geology, and hydrology. One of these pingos is the Lagoon Pingo, which boasts an active spring and a dynamic ecosystem that experiences yearly freeze–thaw cycles and erosion throughout the year. Lagoon Pingo is shaped by groundwater-rich methane fluids pushing upwards through the continuous permafrost, making it a methane source, and enables methane release. During winter the Lagoon Pingo builds up as several dome shaped landforms with icy layers on top. During the summer, the ice melts, the domes collapses and a several crater lakes are generated. Due to the varying moisture levels, sediment grains and methane availability in these pingo crater lakes unique microbial habitats establishes.

In Lagoon Pingo methane fluxes were found to vary between − 0.5 and 1650 nmol m^2^ s^−1^ (Nagel 2020). In our study, two sample sites along a transect (T) were collected, one from the mini source (MS) which had a subsurface water discharge with an elevated methane flux (687.5 nmol m^2^ s^−1^) and one from the still pond (SP), containing water covered locations with no water movements, which exhibited a relatively low methane flux of 1.5 nmol m2/s. Previously a high number of *pmoA* genes relative to the copy number of 16S rRNA genes were found in the still pond was (Nagel 2020), suggesting the potential for high methanotrophic activity which matches our observation of a low methane flux. When analysing the microbial community diversity at these two sites, we found that the alpha diversity was highest in the MS, and the overall bacterial communities were highly diverse within the bacterial domain. Dominant ASVs were affiliated to the phyla *Pseudomonadota, Bacteriodota*, *Gemmatimonadota*, and *Actinomycetota*. The same taxa have also been reported as dominant in studies done across the Arctic and Antarctic marine sediments in addition to studies from lake sediments of the Tibetan Plateau (Xiong et al. 2012; Carr et al. 2015; Müller et al. 2018). The Lagoon Pingo samples were dominated by family *Hydrogenophilaceae*, represented by *Thiobacillus*. *Thiobacillus* is a genus with known chemolithotrophic or mixotrophic bacteria that uses various inorganic electron donors like reduced sulfur compounds and has the ability for carbon fixation by the Calvin-Benson cycle (Hayashi et al. 1999; Orlygsson and Kristjansson 2014). *Thiobacillus* genera are abundant, indicating high sulfur activity and potential denitrification chances at this site.

Within the phylum *Pseudomonadota*, the *Gammaproteobacteria* was the most dominant class in Lagoon Pingo. We observed high abundance of the gammaproteobacterial methanotrophic family *Methylococcaceae* which represented up to 2.5% of the community in MS and only < 1% in the still pond. Among the genera within this family, *Methylobacter* dominated. This genus has shown to be present in many soils on Svalbard, close to Lagoon Pingo (Wartiainen et al. 2003; Høj. et al. 2006; Tveit et al. 2014; Fåne 2020).

Strain LS7-T4A^T^ was successfully isolated and classified in this study, and classified as a *Methylobacter* sp. Its identity was confirmed by 16S rRNA gene- and the *pmoA* gene sequencing, molecular marker genes that can be used for the classification of methanotrophic taxa (Knief 2015). Phylogenetic analysis of the *pmoA* gene revealed that our isolate clusters within *Methylobacter*, indicating that this is a new species within this genus, and further genome analyses revealed clustering with *Methylobacter* sp. isolated from Arctic ecosystems (Fig. 8). The most closely related strain to our isolate is *M. psychrophilus* Z-0021 (Omelchenko et al. 1996) and *M. tundripaludum* SV96^T^ isolated from High Arctic wetland soil, Ny-Ålesund, Svalbard, Norway and was first described in 2006 (Wartiainen et al. 2006), and the genome sequencing of this strain was completed in 2011 (Svenning et al. 2011). This species was identified to have a significant role in the biogeochemistry of Arctic wetland soils emitting methane (Tveit et al. 2023).

Our strain LS7-T4A^T^ has an optimal growth temperature of 13 °C and a maximum growth temperature at 22 °C, which differs from the *M. tundripaludum* SV96^T^, a psychrotolerant strain with optimal growth at 23 °C and maximum growth temperature at 30 °C. Very few psychrophilic methanotrophs, which thrive in low-temperature environments, have been isolated and characterised (Table 3). However, *Methylosphaera hansonii* and *Methylobacter psychrophilus* are two true psychrophilic methanotrophs that have been successfully isolated. *M. hansonii* was found in the surface sediments of an Antarctic meromictic lake (Bowman et al. 1997), while *M. psychrophilus* was isolated from Russian Arctic tundra soil (Omelchenko et al. 1996). Recently, a study conducted in boreal lake ecosystems in Finland reported that the isolate *Methylobacter* sp S3L5C, which based on its characterization and genomic data, is also a psychrophilic methanotroph (Khanongnuch et al. 2022). The dominance of *Methylobacter* sp. in the oxic-anoxic transition zone from boreal and subarctic lakes, ponds, and wetlands is reported through several different studies (Smith et al. 2018; Rissanen et al. 2018; Rissanen et al. 2021; Cabrol et al. 2020) confirming that this is a ubiquitous genus in low temperature environments.

**Table 3:** Comparison of major characteristics of strain LS7-T4A^T^ with other psychrophilic and psychrotolerant species of reported methanotrophs

Characteristics	Strain LS7-T4A^T^	*Methylobacter* sp. strain S3L5C	*Methylobacter psychrophilus* Z-0021^T^	*Methylobacter tundripaludum* SV96^T^		*Methylovulum* sp. strain TFB	*Methylovulum psych rotolerans* Sphl^T^	*Methylosphaera hansonii* ACAM 549^ T^
Cell morphology	Coccoid to rod	Cocci	Rods or cocci	Rods		Cocci, elliptical rod	Cocci	Cocci
*Detection of genes:*								
*pmoA* (pMMO)	+	+	+		+	+	+	+
*mxaF* (MDH)	+	+	+		+	+	−	nr
*mmoX* (sMMO)	−	−	+		−	−	+	−
*nif* H/N2 fixation	+	+	+		+	+	+	+
*cbbL* (RubisCo)	−	−	−		−		−	−
*mauA*	−	−	−		−	−	−	nr
*xoxF*	−	+	+		−	−	−	−
Pigmentation	Light pink	Cream	nr		Pale pink	Transparent	Light pink	− ^a^
Growth temp. (optimal in ° C)	1 − 22 (10 − 15)	0.1 − 20(8 − 12)	1 − 21(3.5 − 7)		5 − 30 (23)	2 − 22 (13 − 18)	2 − 36(20 − 25)	0 − 21 (10 − 13)
pH range	6.4 − 9.3 (7.2 − 8)	6.0 − 8.3(6 − 7.3)	5.9 − 7 (6.7)		5.5 − 7.9	5.2 − 8.5 (6.5 − 7.2)	4 − 8.9 (6 − 7)	7.0
Growth on methanol	+	+	+		− ^b^	+	+	+
Vitamin required	+ / −	nr	+ / −		−	+		− ^c^
Source	Arctic Lagoon Pingo sediments	Lake water layer	Russian Arctic Tundra soil		Wetland soil	Arctic thermal spring	Cold methane seeps and freshwater lake	Antarctic meromictic lake
Reference	This study	Khanongnuch et al. 2022	Omelchenko et al. 1996* and* Rissanen et al. 2022		Wartiainen et al. 2006	Islam et al. 2020	Oshkin et al. 2016	Bowman et al. 1997

^a^Highly purified agar, agarose, and gelrite were likewise failed for pigmentation. ^b^showed poor to no growth on methanol. ^c^Seawater reqired for growth

“ − ” indicates absent and “ + ” indicates present

The genome of *Methylobacter* LS7-T4A^T^ sp. has genes encoding enzymes required for aerobic methane metabolism. Compared with other species, the features of LS7-T4A^T^ are distinguishable, and the average nucleotide identity showed that our strain is a new addition to the genus *Methylobacter*. The GC content also has differences between 0.7 and 5 when compared with other species in the same genus. Methanotrophs obtain energy from oxidation of C1 substrates to CO_2_ and can obtain energy in the form of ATP from oxidative phosphorylation. Our isolate uses methane as a substrate for growth catalyzed by the pMMO enzyme. Type I methanotrophs also have *xoxF*-type pyrroloquinoline (PQQ) dependent methanol dehydrogenase (MDH) genes (Chu and Lidstrom 2016) which were absent in our strain. The genome carries the genes necessary for the synthesis of methanofuran (MFR), and tetrahydromethanepterin (THPMT), which were absent in the recently described Ca. Methylobacter titanis sp. nov (Roldán and Menes 2023). Conversion of formate to CO_2_ is the final methane oxidation stage catalyzed by formate dehydrogenase, which we also found in the genome of our isolate.

The isolate LS7-T4A^T^ uses the RuMP pathway for carbon fixation as most type Ia methanotrophs in the genus *Methylobacter* sp (Collins et al. 2017), but it lacks the enzymes for the serine pathway. Recent studies about *Ca. Methylobacter favarea* B2 (Hogendoorn et al. 2021) and *Ca. Methylobacter titanis* sp. nov (Roldán and Menes, 2023) revealed almost complete serine pathways along with RuMP, which is not typically seen within the genus *Methylobacter* (Chistoserdova et al. 2009). Like most *Methylobacter* species, the genome of our strain lacks RuBisCo (1,5-bisphosphate carboxylase/oxygenase), which was found in the *Methylococcus capsulatus* strain bath (a member of the type Ib *Methylococcaceae*) and some Verrucomicrobial methane oxidizers (Henard et al. 2021; Khadem et al. 2011).

In this study, we have isolated a psychrophilic methane oxidizer belonging to the genus *Methylobacter* in the family Type Ia *Methylomonadaceae*. Relative to *M*. *tundripaludum* SV96^T^, *M*. *psychrophilus* Z-0021^T^, *Methylobacter* sp. S3L5C, ‘Ca. Methylobacter titanis, the *Methylobacter* sp. LS7-T4A^T^ presented in this paper is likely to be distinct species compared to commonly used ANI and dDDH thresholds to distinguish separate species (95% ANI and 70% dDDH; Table S2). Strain LS7-T4A^T^ might have an important role in the biological methane sinks of terrestrial methane seeps such as Lagoon Pingo in Svalbard. Our knowledge of the cold-adapted methane oxidizing bacteria in the open-system pingos is still very limited, yet the results from this work together with the recovered aerobic methanotroph isolate, indicates that the microbial community is important in the methane mitigation in these systems.

### Description of Methylobacter svalbardensis sp. nov

*Methylobacter svalbardensis* (sval.bar.den’sis. N.L. gen pl. n). The local name of a Norwegian archipelago in the Arctic Ocean refers to “the land with the cold coasts.”.

The strain has the following properties: Gram-stain-negative, strictly aerobic and coccoid to rod-shaped cells with a size of 0.8–1.2 × 1.6–2.2 µm. Some cells are motile. Reproduce by binary fission. Colonies are pigmented, light pink, circular and smooth colonies on agar with 1.8 to 2.5 mm in diameter. It is a psychrophilic and obligately methylotrophic strain utilizing methane and methanol via RuMP pathway. Cells do not grow on methylamine, formate, and formaldehyde. Utilise nitrate as a nitrogen source. Vitamins are not required for its growth. Cells contain pMMO and MDH but not sMMO; the genes *xoxF*, *mauA* and *cbbL* are not found in the genome. Contains a *nif*H gene. Growth occurs at 1–22 °C (optimum 10 to 13 °C), at pH 6.4 to 9.3 (optimum pH 7.5 to 8.0). Does not grow on glucose, acetate, succinate, malate, lactate, pyruvate ethanol, methylamine, yeast extract or R2A agar plates. Phylogenetically, strain LS7-T4A^T^ belongs to the genus *Methylobacter* of the family *Methylomonadaceae* Type Ia. The closest present species are *M.* *psychrophilus* Z-0021^T^ (98.95%) and *M*. *tundripaludum* SV96^T^ (99.06%). DNA Genome sequencing of strain LS7-T4A^T^ unveiled a genome length of 4.3 Mbp of 226 contigs with 4272 annotated genes. The G + C content of the DNA is 47.93 mol % from genome. The type of strain LS7-T4A^T^ (DSMZ: 114308; JCM: 39463 ) was isolated from terrestrial methane seep sediments located in Svalbard, Norway.

## Supplementary Information

Below is the link to the electronic supplementary material.

Supplementary file1 (DOCX 516 KB)

Supplementary file2 (XLSX 20 KB)

## Data Availability

The GenBank accession numbers of the16S rRNA genes sequence is OQ832782. The 16S rRNA amplicon Illumina sequence GenBank accession numbers SAMN37693933, SAMN37693934, SAMN37693935, SAMN37693936, SAMN37693937, SAMN37693938, SAMN37693939, SAMN37693940 and draft genome sequence under the BioProject ID: PRJNA1024098 (all sequences will be publicly available after publication). The culture of our isolate is deposited and available in DSMZ with assigned accession number 114308 and JCM with the accession number from 39463.
