# Supplementary material for: Targeting methanotrophs and isolation of a novel psychrophilic Methylobacter species from a terrestrial Arctic alkaline methane seep in Lagoon Pingo, Central Spitsbergen (78° N)
Source: Antonie Van Leeuwenhoek. 2024 Mar 22;117(1):60. doi: 10.1007/s10482-024-01953-1 (PMC10959801; doi:10.1007/s10482-024-01953-1)
Supplement: Supplementary file 1 — Supplementary file1 (DOCX 516 KB) [file 10482_2024_1953_MOESM1_ESM.docx]

**Fig S1:** Relative abundance of bacterial families from sample still pond (SP) and mini source (MS)


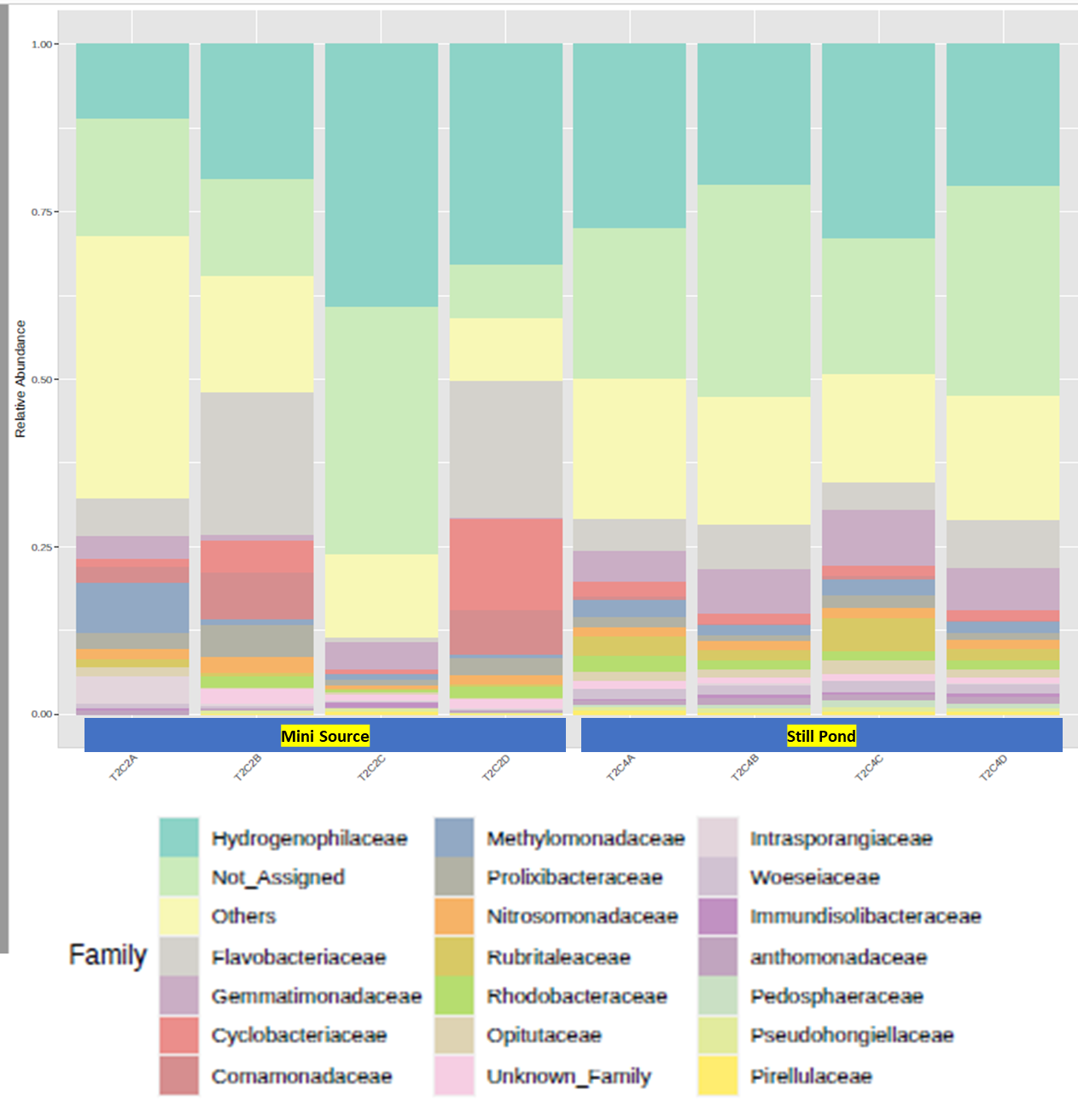


**Fig S2:** Temperature growth curve plotted using Growth rate seen at different temperatures of Methylobacter LS7T4A isolate.

**
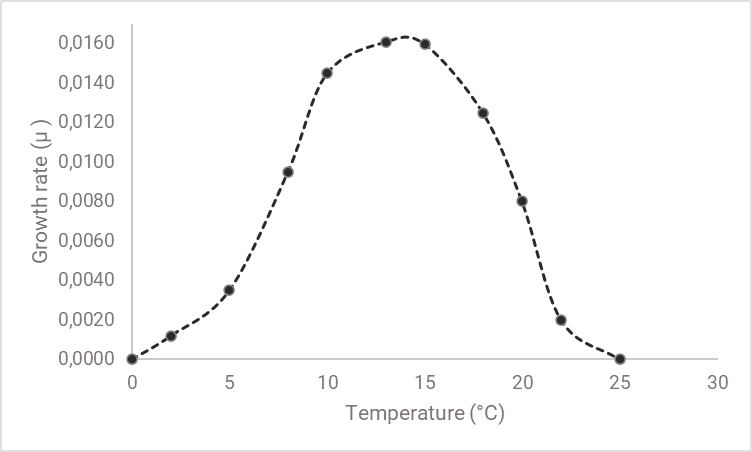
**

**Fig. S3** Results of gene detection of strain LS7-T4A by whole PCR**.**

**Name of primers used:**

**1.** pmoF+ pmoR **2.** A189+mb661 **3.** PmoC617 + A682r **4.** A189 + A682r **5.** *mxaF* (1003f + 1561r) **6.** *mmoX* (882f + 1403r) **7.** *nifH*  **8.** *cbbL* **9.** *manA* **10.** *mmoX*  **11.** PmoF + PmoR (positive control) **12.** *mxaF* **13.** *cbbL* **14.** PmoF + PmoR (negative control)


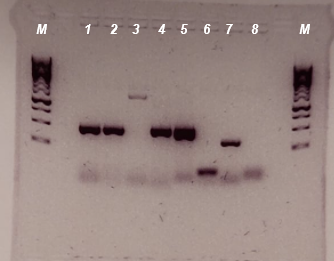

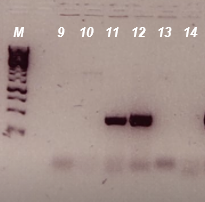


**Table S1**  List of primers, products and results used for whole cells PCR amplification analysis of the strain LS7-T4A.

**Table S2:** Showing measured growth rate at different temperatures from 0 °C to 25°C.

**Table S3:** Comparative table of species from Methylobacter genus with Methylobacter LS7T4A isolate.
